# Gene Expression Profiling of Transcription Factors and Acclimation-Related Genes in *Ribes* spp.

**DOI:** 10.3390/ijms262110367

**Published:** 2025-10-24

**Authors:** Ana Dovilė Zubauskienė, Edvinas Misiukevičius, Vidmantas Bendokas, Emmanuel Gabriel Njoku, Ingrida Mažeikienė

**Affiliations:** 1 Department of Orchard Plant Genetics and Biotechnology, Institute of Horticulture, Lithuanian Research Centre for Agriculture and Forestry, Kauno Str. 30, LT-54333 Babtai, Lithuania; ana.dovile.zubauskiene@lammc.lt (A.D.Z.); edvinas.misiukevicius@lammc.lt (E.M.); vidmantas.bendokas@lammc.lt (V.B.); 2 Department of Biochemistry, Schulich School of Medicine and Dentistry, Western University, 1151 Richmond St, London, ON N6A 3K7, Canada; enjoku3@uwo.ca

**Keywords:** *R. aureum*, *R. hudsonianum*, *R. nigrum*, in vitro, cold stress, deacclimation, reacclimation

## Abstract

The ability of *Ribes* species to survive the fluctuating winter and early spring conditions, relies on the regulation of transcription factors (TFs) and other key genes involved in the abiotic stress response. In this study, we developed specific primers for 33 stress-responsive genes, which may facilitate future functional studies in *Ribes* and other less-characterized lineages within the Saxifragales order. These genes were selected based on a comparative transcriptomic analysis of *R. nigrum* cv. Aldoniai and are known to function in cold acclimation and stress signaling pathways. We analyzed expression profiles of these 33 genes in *R. aureum*, *R. hudsonianum*, and *R. nigrum* microshoot cultures exposed to controlled cold stress, deacclimation and reacclimation treatments. Our results revealed species-specific genetic responses across acclimation cycles of varying durations (24–96 h). Cold stress induces molecular changes in three *Ribes* spp.; however, deacclimation triggered by transient warming significantly reduced freezing tolerance in *R. nigrum*, had a moderate effect on *R. hudsonianum*, and minor impact on *R. aureum*. Gene expression profiling revealed distinct, species-specific regulatory patterns among species during different stress cycles, highlighting conserved and specific genes in acclimation mechanisms within the *Ribes* spp. These findings contribute to a deeper understanding of transcriptional regulation under acclimation cycles in currants and provide molecular tools that may support breeding strategies aimed at enhancing cold tolerance in *Ribes* crops amid increasing climate variability.

## 1. Introduction

Cold stress is one of the most significant environmental challenges faced by plants, especially in temperate regions, where fluctuating temperatures can influence growth, development, and survival of plants. The genus *Ribes* comprises about 150 species, among which *R. nigrum* is the most economically important, with a wide diversity of cultivars and notable adaptation to extreme climates, making it particularly relevant for studies on cold tolerance [1,2]. Wild genotypes such as *R. nigrum* spp. *sibiricum*, *R. dikuscha*, *R. hudsonianum* and their derivatives are regarded as donors of winter hardiness and increased spring frost tolerance of vegetative parts [3].

Individual species of *Ribes* have broad cold hardiness capabilities or tolerance to spring frosts, making this genus an excellent model for studying these regulatory networks [4]. Several studies related to abiotic stress in *Ribes* species have been reported, including cryopreservation [5], acclimation processes [6,7], low-temperature germplasm storage [8] and spring frost [9]. Studies have shown that in blackcurrants, as in other plants, low-temperature stress results in the formation of reactive oxygen species (ROS) and changes in levels of secondary metabolites, phytohormones, amino acids, fatty acids and sugars [5,6,10]. Transcription factors (TFs) are regulatory proteins that bind to specific DNA sequences to control the expression of genes involved in key processes, including stress tolerance, growth, and development [11]. However, no studies using analytical approaches including ‘omics’ tools, have yet provided a comprehensive overview of the gene candidates involved in cold-responsive mechanisms in *R. nigrum*.

Cold stress induces a complex array of molecular, physiological, and biochemical changes in plants [12]. TFs play a central role in plant response cascades to abiotic stress by mediating these responses through binding to specific cis-elements in gene promoters and regulating downstream gene expression. Several key TF families such as AP2/ERF, WRKY, bZIP, bHLH, MYB and NAC have been implicated in mediating plant responses to low temperatures [13,14,15,16]. Each of these families plays a role in mediating the plant’s ability to adapt to low temperatures and cope with environmental stress. These TFs modulate the expression of cold-responsive (*COR*) genes, improve cellular protection mechanisms, and coordinate hormonal and metabolic pathways that contribute to stress adaptation. In the context of cold stress, specific families of TFs such as C-repeat binding factors (CBFs), APETALA2/ethylene-responsive factors (AP2/ERFs), and NACs are crucial for activating cold-responsive genes, enhancing cellular stability, and protecting plant tissues from damage caused by low temperatures [11,17,18]. The WRKY family is known for its role in multiple stress signaling [19], including those from ROS and pathogen response pathways. While the MYB and bHLH families contribute to the regulation of cold-responsive genes and the adaptation of metabolic pathways [11,20,21], they are known to influence anthocyanin biosynthesis and membrane lipid composition under cold stress. Similarly, TFs like AP2/ERF and NAC are important for cold acclimation and regulating the cellular changes that improve plant tolerance to freezing temperatures [11,18], and contribute to the regulation of senescence and programmed cell death during prolonged stress exposure. Other families, such as NF-Y, EIL, and B3, also participate in gene networks that maintain cellular stability and regulate transcriptional reprogramming under adverse environmental conditions [22,23].

High-throughput gene expression analysis using transcriptomic tools enables the identification of cold-responsive genes and regulatory pathways on a genome-wide scale. Such analyses have been successfully applied in model plants and major crops to uncover genes involved in cold acclimation, freezing tolerance, and recovery from stress [24,25,26]. While cold-responsive transcriptomes have been extensively studied in species like *Arabidopsis thaliana*, *Populus* spp., and *Vitis vinifera*, relatively few omics-based studies have been conducted in *Ribes* [24,27,28,29]. Cold stress leads to acclimation, where plants become cold-tolerant. However, plants also undergo a process known as deacclimation, where the cold tolerance developed during exposure to low temperatures is lost when they return to warmer conditions. Reacclimation is the subsequent recovery process, wherein plants regain their cold tolerance. Reacclimation is also a dynamic process that is poorly understood. The molecular mechanisms governing this process are particularly unclear in *Ribes*, where responses to cold stress, deacclimation, and reacclimation may vary significantly across species [4,9].

For examining proteins from different TF families, the study by Mažeikienė et al. [26] provided the foundation for understanding the molecular mechanisms that regulate cold stress tolerance in *R. nigrum*. The de novo transcriptome of *R. nigrum* cv. Aldoniai of interspecific origin (combining the genotypes of *R. nigrum* spp. *europaeum*, *R. nigrum* spp. *sibiricum* and *R. dikuscha*) was assembled to assess the response to biotic stress, but cold stress was also applied to facilitate the spread of virus infection. This previous study opened the way for genetic studies in currants. As a result, the current study aims to explore the expression profiles regulation in different TF families in the three *Ribes* species during cold stress, warm deacclimation, and cold reacclimation in vitro and provides the first report of the *Ribes* spp. response to acclimation processes at the molecular levels. By examining how proteins are expressed during these phases, we aimed to identify key players in acclimation mechanisms. Understanding the transcriptional changes associated with cold acclimation and subsequent deacclimation and reacclimation provides valuable insights into the molecular basis of plant stress adaptation to climate extremes.

## 2. Results

### 2.1. Protein-Protein Interaction (PPI) Network

**Figure 1:**
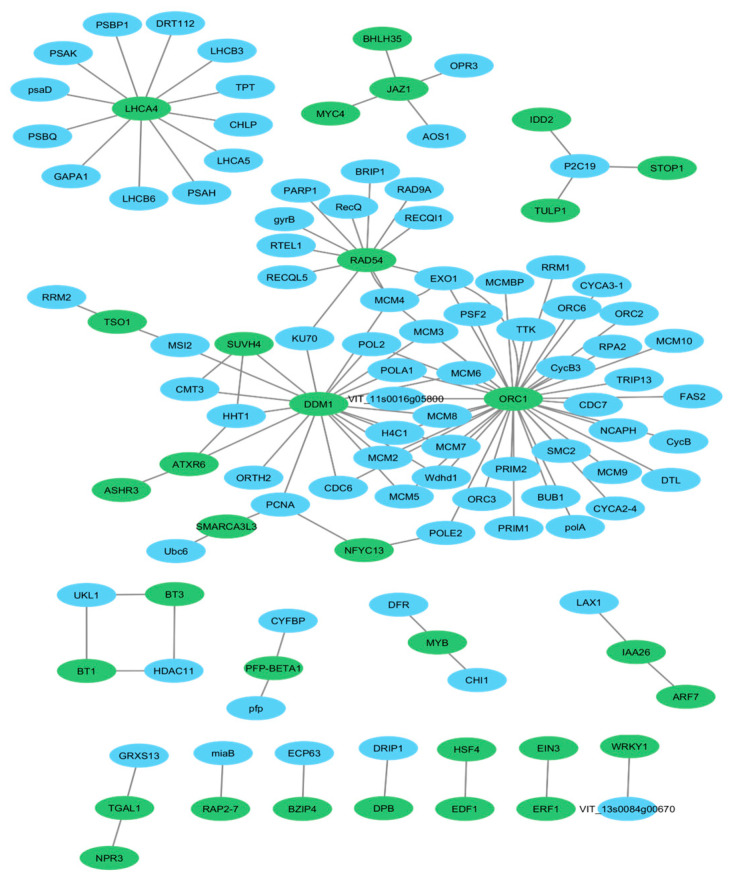
Protein–protein interaction (PPI) network during cold stress (2 vs 4 days) in *R. nigrum* cv. Aldoniai transcriptome. Proteins in green are the key proteins that were selected for detailed gene expression profiling during the cold acclimation, deacclimation and reacclimation experiment. Proteins in blue interact with the key proteins, contributing to broader molecular pathways.

The protein–protein interaction (PPI) analysis was performed using de novo transcriptomic data previously generated from *Ribes nigrum* cv. Aldoniai [26]. A total of 197 differentially expressed genes (DEGs)—71 downregulated and 126 upregulated—were selected for PPI network construction (Table S1). This dataset served as the basis for identifying transcription factors (TFs) and key regulatory proteins based on node degree and betweenness centrality, highlighting hub proteins involved in cold stress response, particularly at the 2-day and 4-day treatment time points (Figure 1).

Among these DEGs, 33 (13 downregulated and 20 upregulated) (green nodes) were identified as putative key proteins in various TF families based on gene annotation and regulatory function prediction (Table S2). Most of these proteins could be transcription factors such as WRKY1, WRKY2, NPR3, JAZ1, BT1, BT3, HSF4, MYB, EIN3, DPB, EDF1, TSO1, STOP1, BZIP4, TGAL1, MYC4, BHLH35, ARF7, ERF1 and RAP2-7. Others involved in chromatin remodeling, such as SMARCA3L3, DDM1, SUVH4, ASHR3 and ATXR6, or in DNA repair such as RAD54 and ATXR6. Notably, several proteins (LHCA4, JAZ1, RAD54, DDM1, and ORC1) appeared as central hubs within the network, interacting with multiple proteins. This suggests their key regulatory roles in the blackcurrant’s low-temperature response and cold stress adaptation (Figure 1).

According to the PPI network we selected 33 proteins (in green), which are members of the 22 different TF families, such as SNF2, bHLH, WRKY, bZIP, MYB and AP2/ERF. (Table S3). These are the key proteins that were selected for detailed gene expression profiling during the acclimation, deacclimation and reacclimation experiment. Other proteins in PPI (in blue) appear to interact with the key proteins, contributing to broader molecular pathways. They might represent downstream or upstream components in cold stress response, cellular repair, chromatin remodeling, or other processes like DNA repair, cell cycle regulation, and transcriptional regulation. The largest TF family SNF2 was composed of 3 proteins DDM1, SMARCA3L3 and RAD54. This family contributes significantly to cold stress responses by remodeling chromatin for the activation of cold-responsive genes, including *CBF*s (C-repeat binding factors) and *COR* (cold-regulated) and by interfacing with other regulatory pathways. To validate transcript abundance, specific oligonucleotide primers were designed (Table S3) for TFs and cold stress-response genes represented by the green nodes in the PPI network, and their expression levels were further analyzed using qRT-PCR (Figure S1).

### 2.2. Hierarchical Clustering of Transcription Factors in Response to Hardening

**Figure 2:**
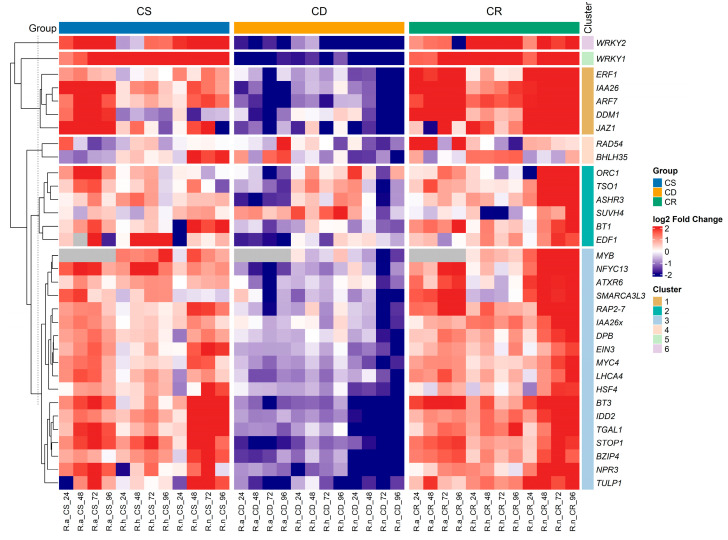
Heatmap profile of expressed genes under different treatment stages: cold stress (CS), cold deacclimation (CD) or cold reacclimation (CR) in *R. aureum* (R.a.), *R. hudsonianum* (R.h.) and *R. nigrum* (R.n.) microshoots. In the scale bar, blue indicates low (−2) gene expression value, and red indicates high expression (2).

Heatmap displays expression profiles across individual samples of treated blackcurrant species, with 33 genes listed on the y-axis and samples on the x-axis (Figure 2). Expression data were log_2_-transformed and normalized prior to visualization (Figure S1). The color scale represents relative expression changes, where red indicates upregulation, blue—downregulation and white—no significant change. Treatment groups are annotated with colored bars above the heatmap: blue for cold stress (CS), yellow for cold deacclimation (CD), and green for cold reacclimation (CR).

In general, most blackcurrant transcription factors displayed upregulated expression under cold stress (CS) and cold reacclimation (CR) treatments. In contrast, a marked decrease in expression levels was observed during cold deacclimation (CD) in *Ribes* species (Figure 2). Comparative analysis across species showed that *R. nigrum* demonstrated the greatest intra-specific variation in gene expression levels, particularly under the cold reacclimation (CR) treatment.

The heatmap revealed six hierarchical clusters, highlighting diverse transcriptional patterns and suggesting functional specialization among genes studied. The largest cluster contains 18 genes such as *MYB*, *NFYC13*, *ATXR6*, *SMARCA3L3* and *RAP2-7*, reflecting a broad regulatory module that likely integrates multiple stress-responsive and hormonal signaling pathways. Members of this cluster, including *MYB* and *RAP2-7*, are known to regulate responses to abiotic stresses such as cold and oxidative stress, while *ATXR6* and *SMARCA3L3* suggest epigenetic regulation through chromatin remodeling. Together, these distinct clusters highlight the complexity of transcriptional reprogramming in blackcurrant during cold stress and acclimation, illustrating a finely coordinated yet functionally diverse network of transcription factors that underpins environmental adaptation. The second cluster, comprising *ORC1*, *TSO1*, *ASHR3*, *SUVH4*, *BT1*, and *EDF1*, is associated with chromatin remodeling and cell cycle regulation, suggesting roles in genome stability and stress-induced gene expression. Members of the third cluster *ERF1*, *IAA26*, *ARF7*, *DDM1*, and *JAZ1* are collectively involved in hormone signaling and epigenetic regulation, critical for stress-responsive transcriptional reprogramming. The fourth cluster, containing *RAD54* and *BHLH35*, points to functions in DNA repair and transcriptional regulation during cold stress, essential for maintaining genomic integrity and activating acclimation-related genes. The fifth and sixth clusters consist of *WRKY1* and *WRKY24*, respectively, highlighting key roles of WRKY family TFs in cold stress signaling and defense regulation.

**Figure 3:**
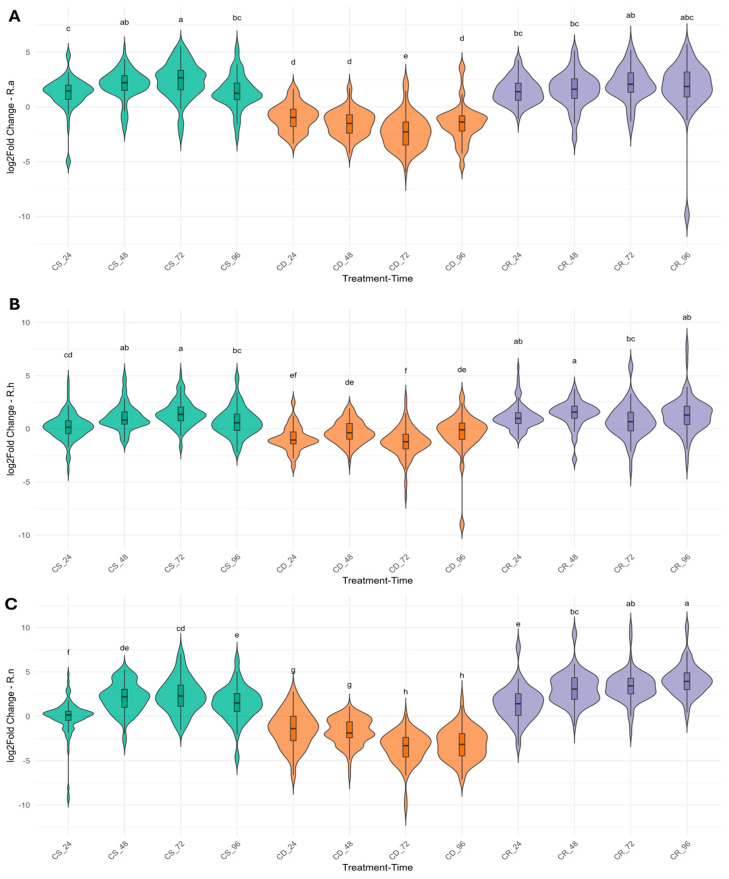
Violin plots depict the gene expression levels of *Ribes* species under different treatment stages: cold stress (CS), cold deacclimation (CD) or cold reacclimation (CR). (**A**) *R. aureum*, (**B**) *R. hudsonianum*, (**C**) *R. nigrum*. Different letters represent statistical reliability within a species (*n* ≥ 3).

Violin plots were used to visualize the expression patterns of 33 genes in various TF families across treatment stages—CS, CD, and CR in *R. aureum* (A), *R. hudsonianum* (B), and *R. nigrum* (C) (Figure 3). Each plot reveals both interspecific and treatment-dependent differences in expression variability and central tendencies. Likewise, this analysis supports the heatmap results (Figure 2), demonstrating consistent TFs and acclimation-response genes expression trends across the experimental stages. These patterns suggest a potential role for selected TFs and genes in initiating and maintaining cold-responsive transcriptional programs.

In *R. aureum* (Figure 3A), expression levels were markedly upregulated during the CS stage, with a broad distribution indicative of high transcriptional activity and variability, particularly after 24 h. Upon transition to CD, a notable decrease in gene expression was observed, reflecting the downregulation of genes encoding cold-responsive proteins and TFs. During the CR stage, expression levels increased again but did not fully return to CS levels, suggesting a partial reactivation of cold-responsive transcriptional pathways. Additionally, high variability in gene expression was observed at the final time point of the experiment (CR_96), indicating dynamic transcriptional regulation during prolonged cold exposure.

In *R. hudsonianum* (Figure 3B), the expression of genes also showed an initial upregulation under CS, although overall expression levels were lower compared to *R. aureum* and *R. nigrum*. This pattern suggests an active but relatively moderate early transcriptional response to cold exposure. A pronounced downregulation was observed during CD, consistent with the suppression of stress-related transcriptional activity. The most significant deviations, characterized by a substantial reduction in the expression of several genes, were observed at the CD_72 and CD_96 time points. During the CR stage, expression levels moderately increased but exhibited greater variability, particularly at the later time points—CR_72 and CR_96, indicating a dynamic reactivation of transcriptional regulation in response to renewed cold stress.

In *R. nigrum* (Figure 3C), while upregulation of TFs and acclimation-response genes was evident during the CS stage, the distribution of expression levels was more heterogeneous than in the other species, suggesting a more diverse or flexible transcriptional response. The highest expression variability was observed at the CS_24 time point. A substantial decline in gene expression occurred during the CD phase, particularly at CD_72, followed by a partial recovery during the CR stage, indicating a moderate reactivation of cold-responsive transcriptional pathways.

### 2.3. Deacclimation Impact on Genes Expression for Hardening in Ribes spp.

**Figure 4:**
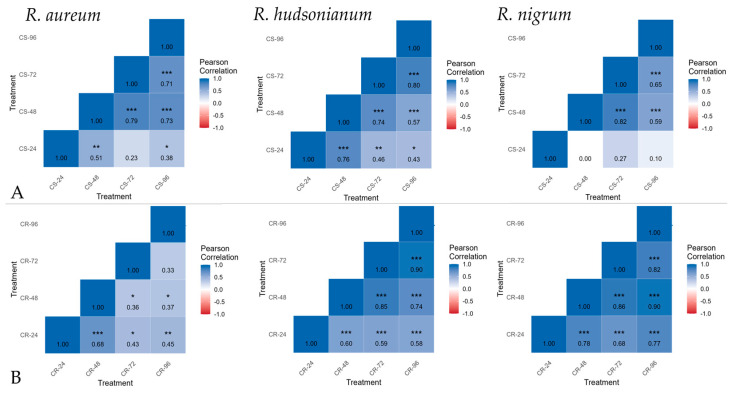
Expression consistency of TFs and acclimation-related genes across timepoints during CS—cold stress (**A**) and CR—cold reacclimation (**B**) hardening experiments in *R. aureum*, *R. hudsonianum* and *R. nigrum* (* *p* value = 0.05, ** *p* value <0.05, *** *p* value = 0.00).

Our correlation analysis revealed distinct differences in the temporal stability of TFs and acclimation-response genes expression among the three *Ribes* species under first CS and second CR hardening regimes. These differences suggest genotype-specific strategies for maintaining transcriptional homeostasis during cold adaptation (Figure 4).

In *R. aureum* genes expression stability was largely confined to the later stages of CS hardening, where significant correlations were observed between 48–96 h periods. This suggests that *R. aureum* gradually establishes a consistent transcriptional program during sustained exposure to subzero temperatures. In contrast, under CR, reliable correlation was observed only at the early stage (24–48 h), after which coordinated expression patterns diminished. Thus, *R. aureum* appears more reliant on a prolonged, cumulative acclimation process (CS), while its rapid response to abrupt cooling (CR) after a warm deacclimation period (CD) is less stable and transient.

By comparison, both *R. hudsonianum* and *R. nigrum* microshoots displayed robust transcriptional stability under CS and CR treatments. In *R. hudsonianum*, significant correlations were maintained across nearly all sampling intervals during CS, and consistently across all periods during CR. Similarly, *R. nigrum* microshoots showed reliable correlations across late CS stages and throughout CR. These findings indicate that *R. hudsonianum* and *R. nigrum* possess more flexible and resilient transcriptional programs, enabling stable expression of TFs and acclimation-related genes during both acclimation and reacclimation under abrupt cooling. Notably, *R. hudsonianum* demonstrated the broadest and most consistent stability pattern, suggesting a greater adaptive genetic capacity to maintain coordinated acclimation-response networks among the three studied species.

Our results highlight genotypic differences in the dynamics of TFs and acclimation-related genes expression stability under hardening conditions. *R. aureum* appears specialized for gradual cold acclimation, with limited stability under abrupt cooling. In contrast, *R. hudsonianum* and *R. nigrum* exhibit broad transcriptional resilience, suggesting that they maintain functional stress-response networks across diverse hardening regimes. Such differences may reflect evolutionary adaptation: *R. aureum* may rely on gradual seasonal transitions, whereas *R. hudsonianum* and *R. nigrum* possess stronger mechanisms for coping with variable or rapid temperature fluctuations.

## 3. Discussion

This study provides the first comprehensive transcriptomic analysis of acclimation-related genes and transcription factors (TFs) dynamics in *Ribes* species during cold stress, cold deacclimation, and cold reacclimation abbreviated as CS, CD, and CR, respectively. The integration of protein–protein interaction (PPI) network construction, hierarchical clustering, and differential expression profiling revealed a core set of 33 proteins from 22 TF families that play central roles in cold stress. Twenty-one TFs from the PPI network were selected for analysis of acclimation cycles due to their role in direct regulation of gene expression through DNA binding. They include families such as WRKY, MYB, bHLH, ERF, JAZ, bZIP, and EIN3. The remaining investigated twelve acclimation-related genes are involved in chromatin remodeling, signal transduction, or other regulatory processes, indirectly influencing transcription but not acting as direct transcription factors. Our findings emphasize the regulatory complexity of acclimation phases in *Ribes* spp. microshoots extending the current knowledge on plant cold stress adaptation mechanisms. TFs and acclimation-related genes activation patterns in our research reflect conserved cold-response pathways in *Ribes* spp. Transcriptomic profiling showed a strong transcriptional activation of selected genes and TFs during cold stress, followed by widespread repression during deacclimation and partial reactivation during reacclimation according to expression data. These sequential regulation profiles are similar to cold acclimation processes in *Arabidopsis thaliana*, where deacclimation is associated with rapid transcriptomic shifts favoring growth resumption, while reacclimation re-establishes freezing tolerance [17,30,31]. The upregulation of AP2/ERF, NAC, WRKY, MYB, and bHLH families in *Ribes* species aligns with their well-established functions in activating *COR* (cold-regulated) genes, stabilizing membranes, and modulating metabolic adaptation under low temperatures [17,30,32,33,34,35,36]. In *Ribes* spp., the dynamics of cold deacclimation and the potential to reacclimate in flower buds of *R. nigrum* and *R. rubrum* were investigated. The study found that during deacclimation flower buds lose their freezing tolerance which poses risks to fruit yield under winter warming [2,6,37]. Similar expression patterns have been reported in perennial crops such as paper mulberry, strawberry, grapevine, and peach, where deacclimation represents a key stage of vulnerability to fluctuating climates [38,39,40,41].

Chromatin remodeling emerges as a central regulatory layer during acclimation, deacclimation and reacclimation in *Ribes*. A notable finding is the enrichment of chromatin remodelers (SNF2 family members *DDM1*, *RAD54*, *SMARCA3L3*, and *ORC1*) as hubs in the PPI network. Chromatin accessibility has been shown to be critical for activating CBF-dependent transcriptional cascades in cold response [42,43]. In plants, SWI/SNF-like ATP-dependent remodelers regulate gene responsiveness to temperature changes by modulating nucleosome positioning [44]. Our data suggests that similar mechanisms are conservative in *Ribes* spp., particularly given the prominence of DNA repair and replication regulators *ORC1* and *RAD54* in stress-induced transcriptional programs. Epigenetic regulation through histone modification, DNA methylation, and chromatin remodeling is increasingly recognized as essential for cold stress memory [42,43], and our study extends this concept to woody perennials.

Crosstalk of hormone TFs underlying stress and developmental regulation during stress were obtained. The clustering of *ERF1, JAZ1*, *IAA26*, and *ARF7* indicates that hormonal regulation is deeply integrated with cold signaling in *Ribes*. Hormone-responsive TFs modulate the trade-off between growth and stress tolerance, particularly during deacclimation when energy allocation shifts toward developmental processes [45]. Jasmonate, auxin, and ethylene signaling networks are known to fine-tune responses to fluctuating temperatures [18,46,47]. The observed transcriptional suppression of hormone-associated genes and TFs during CD and their partial reactivation during CR suggest a regulatory checkpoint that prevents premature growth initiation while enabling recovery when cold returns.

Our study revealed notable species-specific plasticity in the expression profiles of *Ribes* species under acclimation cycles, particularly in response to cold acclimation and reacclimation. This plasticity underscores the interspecific breeding potential in *Ribes* hybrids, highlighting the opportunity to exploit genetic diversity for improved cold tolerance in future cultivars. Among the three *Ribes* species (*R. nigrum*, *R. aureum*, and *R. hudsonianum*) examined, *R. nigrum* exhibited the higher gene expression variability, especially during reacclimation after exposure to cold stress.

Given that cold tolerance in blackcurrant is critical for its commercial cultivation, the transcriptional responses we observed may be indicative of adaptive traits that have evolved over time. This adaptability is essential for breeding new cultivars with enhanced resilience to winter thaws and spring frosts, a phenomenon that has become increasingly relevant with climate change. The intraspecific variation in chilling requirements among *R. nigrum* cultivars provides a rich genetic resource for precise breeding strategies, offering the potential for improved cultivars that can withstand unpredictable climatic fluctuations. In our study, *R. nigrum* (blackcurrant) displayed the most dynamic transcriptional responses, with the most prominent changes observed during reacclimation. This variability may reflect ecological plasticity—the ability of *R. nigrum* to adapt to a wide range of environmental conditions. *R. nigrum* has long been known for its broad ecological adaptation, particularly in the context of cold tolerance. The well-established winter chill requirements for blackcurrant cultivars and strong cultivar-specific variation in chilling responses [2,48,49] suggest that different genotypes may possess distinct mechanisms for handling cold stress. This transcriptional plasticity may be a result of these genetically based variations, enabling *R. nigrum* to respond flexibly to environmental changes, particularly in regions with variable winter chill and spring frost patterns. This flexibility likely reflects the plant’s long history of breeding for cold tolerance, supporting its use in both temperate and subarctic climates [1,3].

When comparing *R. nigrum* to the other two species, *R. aureum* exhibited a strong and early transcriptional response, while *R. hudsonianum* showed a more moderate profile, consistent with its native distribution in colder regions. This early response suggests that *R. aureum* may have evolved mechanisms to quickly respond to environmental changes, possibly as an adaptation to colder environments where sudden temperature fluctuations are common. These interspecific differences may serve as a valuable genetic resource for developing cultivars resilient to unpredictable winter thaws and spring frosts [3].

Evolution may have shaped these PPI network dynamics differently among genotypes. Drawing parallels from studies in *Arabidopsis* ecotypes, genotypic variation in C-repeat binding factor (CBF) regulatory elements correspond with differing acclimation capacities and freezing tolerance [50,51]. Our results suggest *R. hudsonianum* and *R. nigrum* have evolved the regulatory circuits of genes in various TF families more resiliently, enabling stable expression across temporal scales and stress types. In contrast, *R. aureum* may require longer or more predictable cold exposure to establish effective acclimation networks. Similarly, candidate gene regulation during cold hardening in species like *Prunus mume* highlights chromatin accessibility and transcriptional rewiring as key mechanisms underlying variation in tolerance [52]. Integrating knowledge of PPI network stability may guide marker-assisted selection or molecular breeding strategies to develop cultivars with more generalized stress tolerance.

From a practical standpoint, these insights have implications for breeding and selection of cold-tolerant *Ribes* cultivars. *R. hudsonianum* and *R. nigrum* genotypes like cv. Aldoniai with transcriptional stability under both acclimation and reacclimation periods, may serve as valuable genetic resources for improving stress tolerance in breeding programs, particularly under conditions of climate variability. Our findings emphasize that cold response in *Ribes* is governed by multi-layered transcriptional regulation involving chromatin remodeling, hormonal integration, and stress-specific proteins and TFs’ networks. This knowledge provides a foundation for targeted breeding using molecular markers associated with key proteins and TFs. Functional validation through CRISPR/Cas9 or overexpression studies could further elucidate gene function. Additionally, integrating transcriptomics with metabolomics and epigenomic profiling will be essential for fully mapping acclimation and stress memory networks [22,32,35,42]. Such multi-omics approaches are particularly valuable in perennial fruit crops where climate-driven temperature fluctuations pose significant production risks. This research will contribute to advancing breeding strategies for enhancing plant adaptability to climate change and improving cold stress resilience in berries crops.

## 4. Materials and Methods

### 4.1. Plant Material and Stress Conditions

This study combined previously generated transcriptomic data with newly obtained data from additional low-temperature treatments. The core dataset was derived from our earlier RNA-seq analysis of *Ribes nigrum* cv. Aldoniai, performed by Novogene [26], which served as the basis for Tables S1 and S2, and Figure 1. Although the de novo assembly focused on biotic stress, cold stress was also applied (+4 °C for 2 and 4 days), allowing the dataset to reflect abiotic responses in blackcurrants as well.

**Figure 5:**
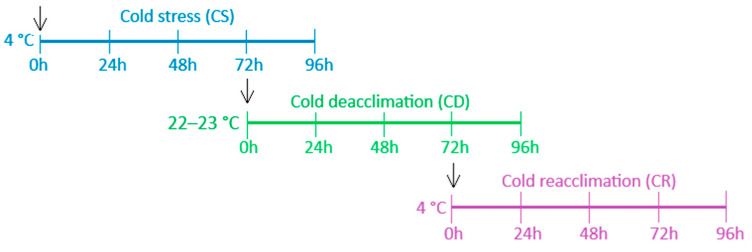
Experimental design containing cold stress (CS), cold deacclimation (CD), and cold reacclimation (CR) processes. Arrows indicate control points of the experiment at each stress stage.

In the present study, this transcriptome was re-analyzed with a focus on acclimation-response genes from various transcription factor (TF) families related to cold stress and applied to a broader range of genotypes. Microshoots of *R. aureum*, *R. hudsonianum* and *R. nigrum* having different winter hardiness were grown under in vitro conditions in the growth chamber at the Department of Orchard Plant Genetics and Biotechnology, Institute of Horticulture, LRCAF, according to the methodology and conditions described by Juškytė et al. [53]. Experiment design were performed according to scheme shown in Figure 5.

At each point (0, 24, 48, 72 and 96 h), three individual plants from the treatment group (cold stress (CS), cold deacclimation (CD), and cold reacclimation (CR)) were sampled to ensure an adequate number of biological replicates. To control potential aging or time-dependent effects unrelated to temperature treatment, microshoots were sampled from an untreated control group at each corresponding time point for each treatment phase. The control for each treatment stage consisted of microshoots indicated by black arrows in Figure 5. These time-matched controls enabled differentiation between gene expression changes specifically induced by temperature regimes and those arising from developmental progression during the 10-day in vitro culture period.

All samples were frozen in liquid nitrogen and stored at −80 °C for RNA extraction.

### 4.2. RNA Extraction and qRT-PCR Analysis

Total RNAs were isolated from *R. aureum*, *R. hudsonianum* and *R. nigrum* microshoots using GeneJET Plant RNA Purification Mini Kit (Thermo Scientific, Vilnius, Lithuania) following the manufacturer’s instructions. The concentration and quality of the RNAs were measured using NanoDrop Implen GmbH spectrophotometer (Implen, Munich, Germany). Isolated RNA was employed to synthetize a first-strand cDNA by Maxima H Minus First Strand cDNA Synthesis Kit (Thermo Fisher Scientific, Waltham, MA, USA). The quantitative real-time PCR (qRT-PCR) on three biological replicates was performed using the PowerUp™ SYBR™ Green Master Mix (Thermo Fisher Scientific, Waltham, MA, USA) with standard thermo cycling mode (60 °C for annealing and extension) using Applied Biosystems QuantStudio 5 (Thermo Fisher Scientific, Waltham, MA, USA). Specific oligonucleotide primers (Table S3) for 33 genes encoding cold-response proteins and TFs and 1 reference gene (Actin) were designed with the Primer3web v4.1.0. TFs of *R. nigrum* cv. Aldoniai with significant expression levels were selected from de novo transcriptome (raw data are available on BioProject PRJNA797914 in the NCBI database).

### 4.3. Statistical Analysis and Data Visualization

Protein–protein interaction (PPI) analysis was performed using predicted protein sequences derived from the *R. nigrum* cv. Aldoniai de novo transcriptome [26]. The interaction data for PPI network was retrieved from the STRING database and visualized using Cytoscape v3.10.1 [54].

Heatmap and violin plot graphs represent gene expression data of selected genes from various TF families assessed by the 2 ^−ΔΔC^T method in *R. aureum, R. hudsonianum* and *R. nigrum* in three biological replicates [55]. Gene expression data were processed and visualized using R v4.3.2, employing various packages from the Comprehensive R Archive Network (CRAN). The data were imported via the readxl package [56] and subsequently cleaned and reshaped using dplyr [57], tidyr [58], and tibble [59]. ^Δ^Ct values were calculated by subtracting the values of the reference gene Actin. For the creation of heatmaps, mean log_2_ Fold Change (log_2_FC) values were aggregated based on genotype, treatment, and time point and visualized using ComplexHeatmap v2.25.2 [60], along with hierarchical clustering techniques. Custom color palettes were created from the RColorBrewer v1.1-2 [61]. Additionally, gene-level boxplots and genotype-specific violin plots were developed using ggplot2 [62]. Statistical comparisons were conducted using ANOVA, followed by Tukey’s HSD test, as implemented in the Agricolae package v1.3-5 [63], with grouping letters prominently displayed on the corresponding plots. Pearson correlation was calculated between the expression data log_2_FC among CS and CR treatments with correlation coefficients and significance levels (*p* ≤ 0.05) using cor, cor.test and visualized using ggplot2 [62].

## 5. Conclusions

This study provides the first transcriptome-wide analysis of differential expression of the acclimation-related genes in *Ribes* species (*R. aureum*, *R. hudsonianum* and *R. nigrum*) during three acclimation cycles—cold stress, warm deacclimation, and cold reacclimation phases. Based on transcriptome sequencing data and protein–protein interaction network construction, 33 key genes belonging to 22 transcription factor (TF) families were identified. These genes highlight chromatin remodelers (SNF2 family) and hormone-related regulators as central hubs in cold stress adaptation mechanism. Cold stress strongly activates transcriptional programs in *Ribes* microshoots, whereas deacclimation induces widespread downregulation, followed by partial transcriptional recovery during reacclimation. Epigenetic regulators, particularly chromatin remodelers such as *DDM1*, *RAD54*, and *SMARCA3L3*, play a pivotal role in the transcriptional reprogramming underlying cold acclimation. Hormone-responsive TFs (*ERF*, *JAZ1*, *ARF7*) coordinate stress signaling with developmental control, underlining the complexity of cold response regulation. *R. nigrum* displays greater transcriptional plasticity than *R. aureum* and *R. hudsonianum*, suggesting a higher potential for adaptation to fluctuating environmental conditions. The plasticity in cold stress responses observed in our study also provides an opportunity to fine-tune hybrid breeding strategies. Crossing *R. nigrum* with *R. aureum* could combine the rapid cold response of *R. aureum* with the flexible acclimation profiles of *R. nigrum*, potentially creating hybrids that offer both early cold tolerance and long-term adaptability. Similarly, hybrids with *R. hudsonianum* could offer greater resilience to extreme cold, particularly in regions subject to deep freezes and sudden temperature swings.

Overall, this research advances our understanding of the molecular mechanisms of cold stress tolerance in the *Ribes* genus. Transcriptomic analyses could help identify the key genes responsible for cold tolerance and acclimation plasticity, providing a more targeted approach for hybrid development. Additionally, studies that integrate phenotypic data, such as growth patterns, yield, and cold hardiness in field conditions, will be crucial for determining the real-world applicability of these transcriptional responses in breeding programs. The data generated provides a foundation for marker-assisted breeding and genetic improvement of cold-hardy berry cultivars, with relevance under climate variability. Future studies should focus on functional characterization of key genes and TFs, epigenetic modifications, and multi-omics integration to fully decipher acclimation and reacclimation networks.

## Data Availability

Data is contained within the article, Supplementary Materials and in NCBI database.

## Supplementary Materials

The following supporting information can be downloaded at: https://www.mdpi.com/article/10.3390/ijms262110367/s1.
