# Supplementary material for: Gene Expression Profiling of Transcription Factors and Acclimation-Related Genes in Ribes spp
Source: Int J Mol Sci. 2025 Oct 24;26(21):10367. doi: 10.3390/ijms262110367 (PMC12609403; doi:10.3390/ijms262110367)
Supplement: Supplementary file 1 [file ijms-26-10367-s001.zip › ijms-3899103-supplementary/Figure_S1.pdf]

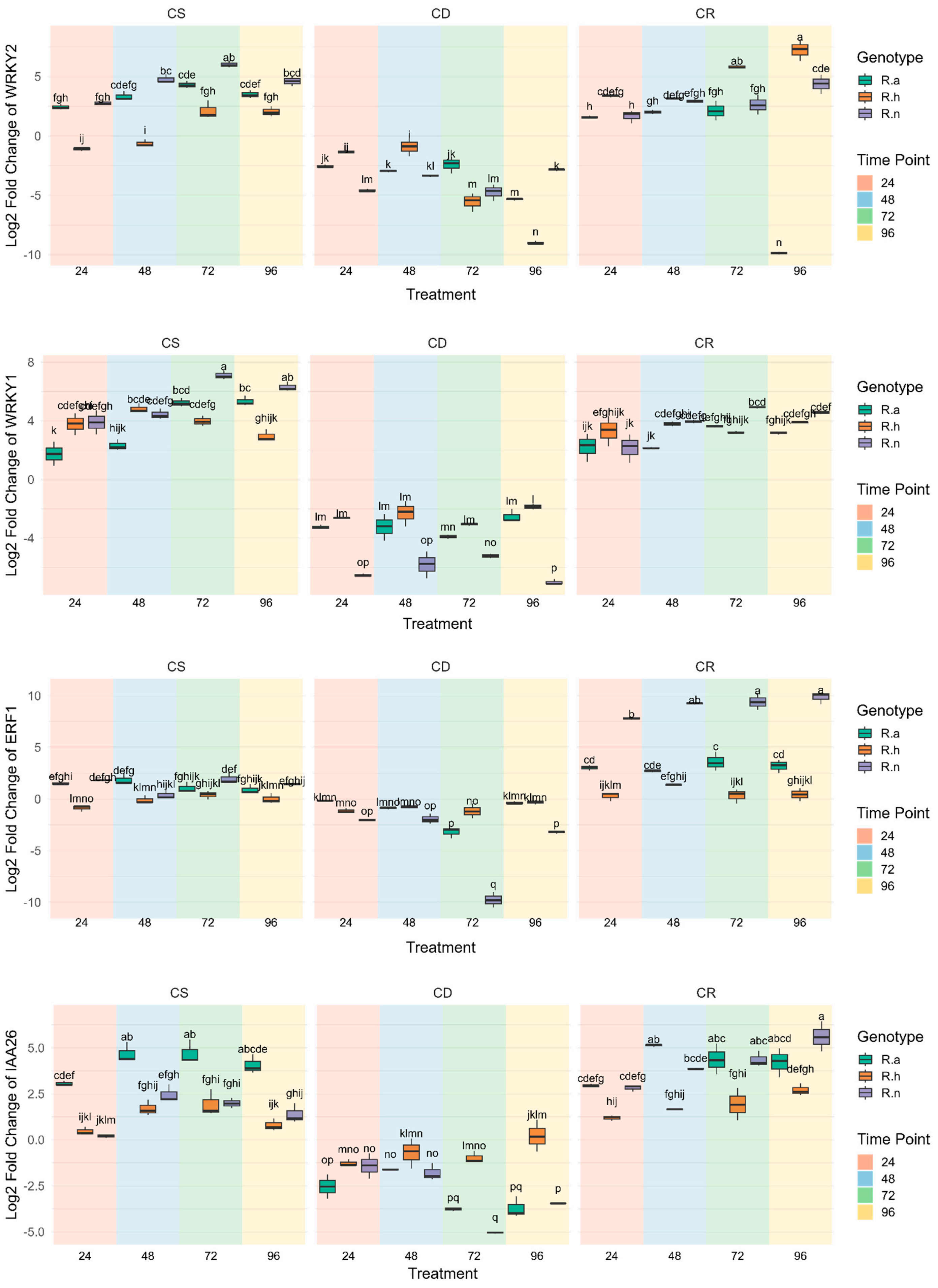

CS
CD
CR
Log2 Fold Change of WRKY2
Treatment
24
48
72
96
Genotype
R.a
R.h
R.n
Time Point
24
48
72
96
Log2 Fold Change of WRKY1
Log2 Fold Change of ERF1
Log2 Fold Change of IAA26

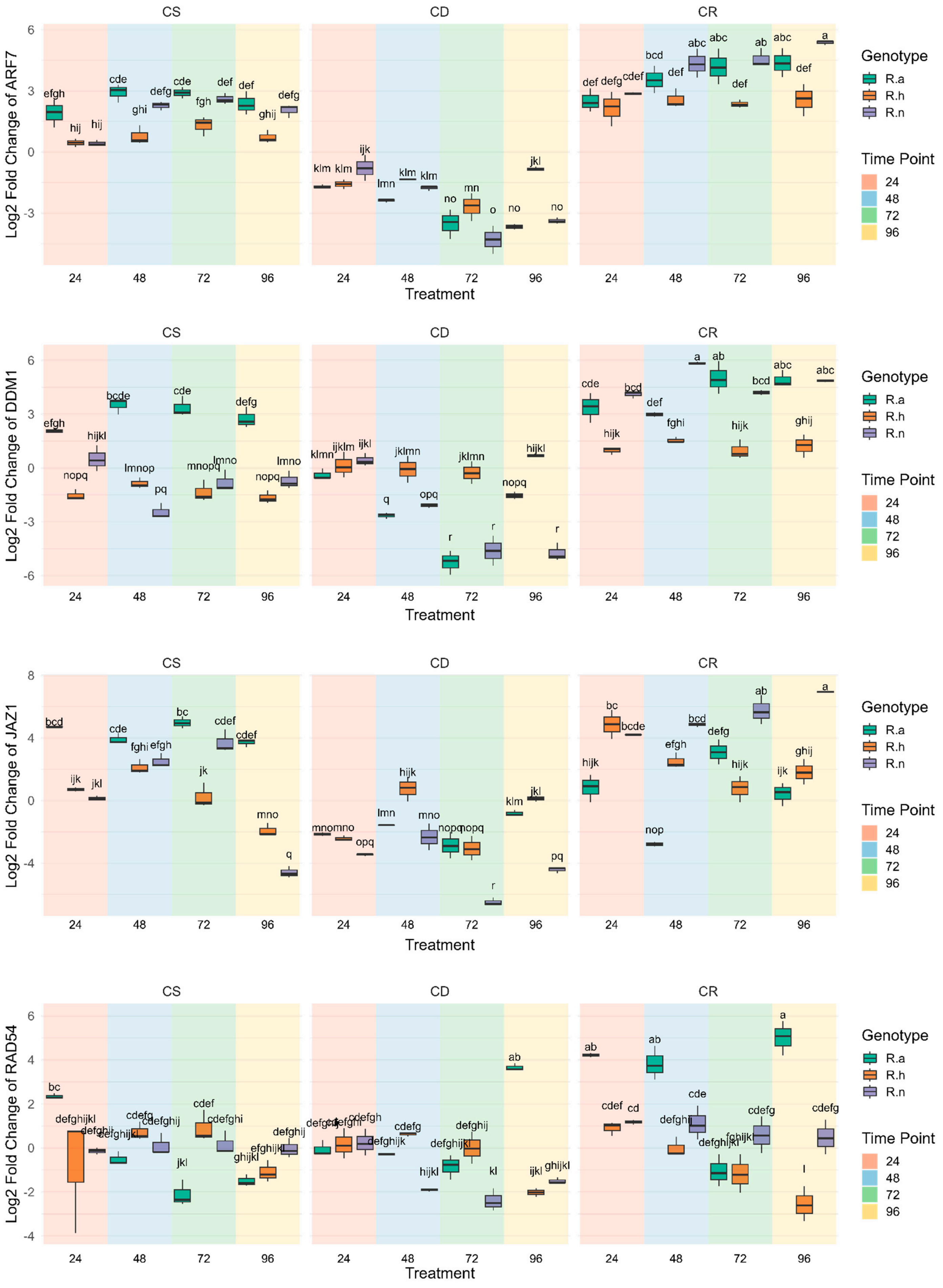
CS
CD
CR
Log2 Fold Change of ARF7
Log2 Fold Change of DDM1
Log2 Fold Change of JAZ1
Log2 Fold Change of RAD54
Treatment
24
48
72
96
Genotype
R.a
R.h
R.n
Time Point
24
48
72
96

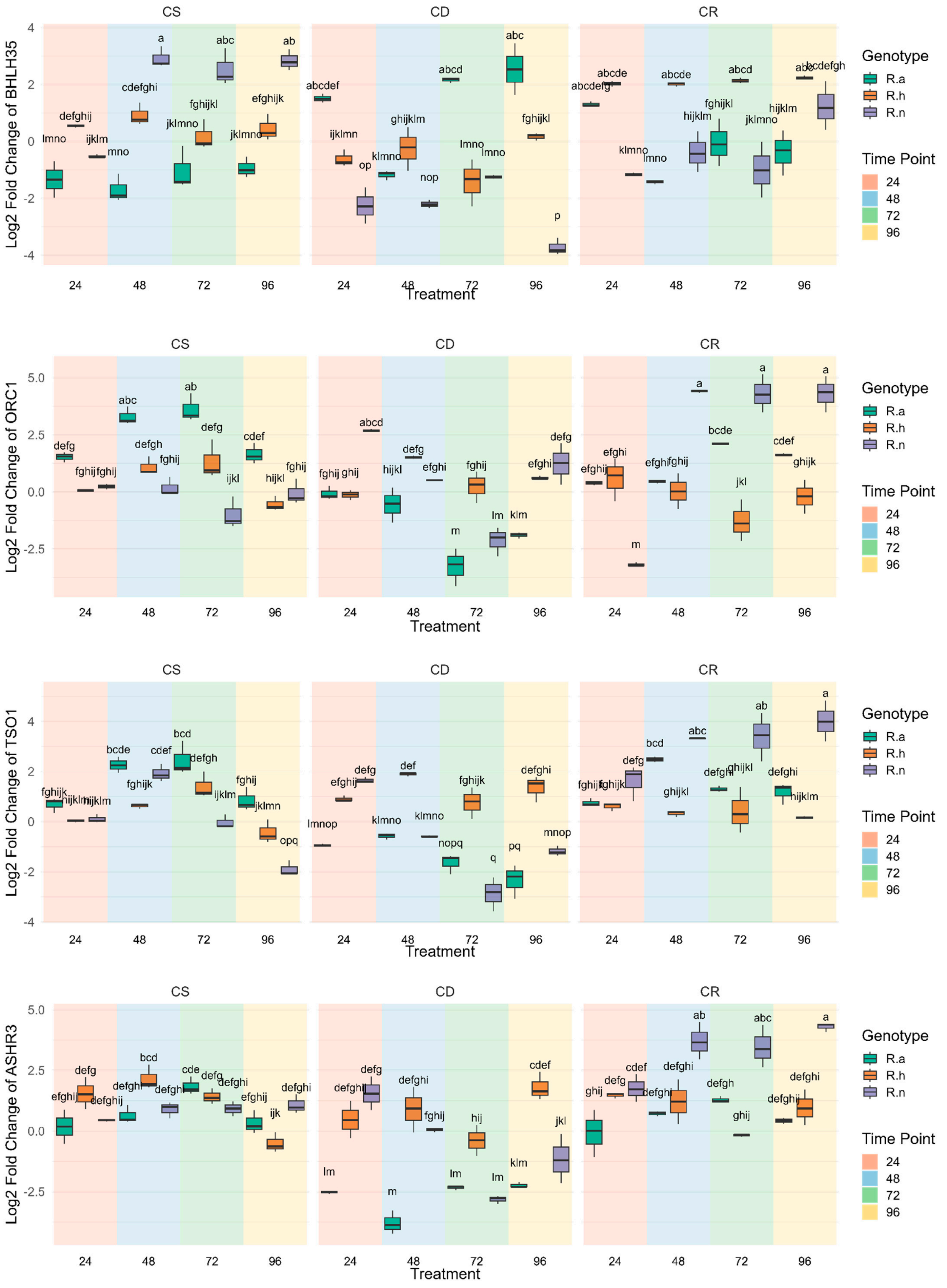

CS
CD
CR
Log2 Fold Change of BHLH35
Log2 Fold Change of ORC1
Log2 Fold Change of TSO1
Log2 Fold Change of ASHR3
Treatment
24
48
72
96
Genotype
R.a
R.h
R.n
Time Point
24
48
72
96

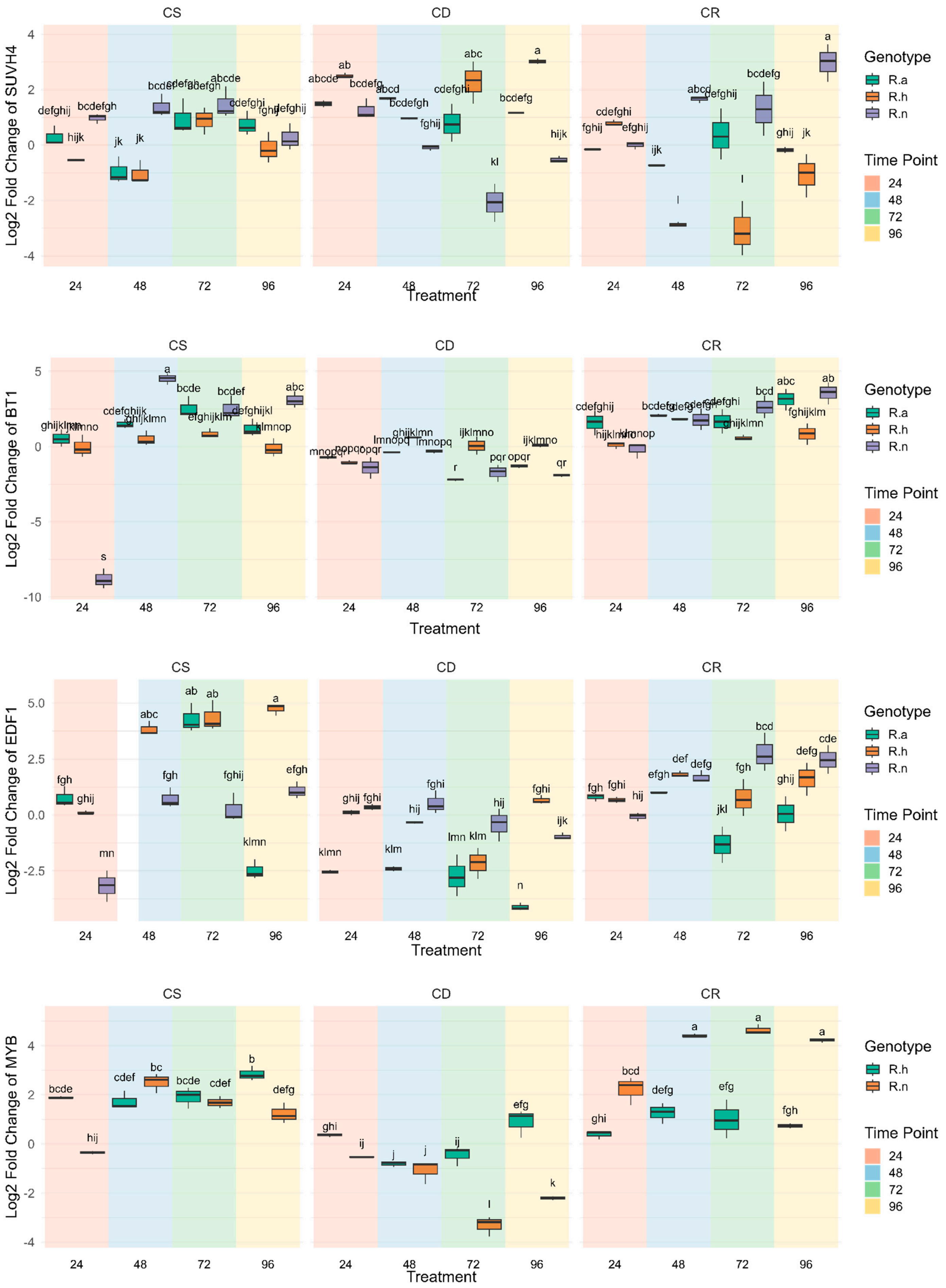
CS
CD
CR
Log2 Fold Change of SUVH4
Log2 Fold Change of BT1
Log2 Fold Change of EDF1
Log2 Fold Change of MYB
Treatment
24
48
72
96
Genotype
R.a
R.h
R.n
Time Point
24
48
72
96

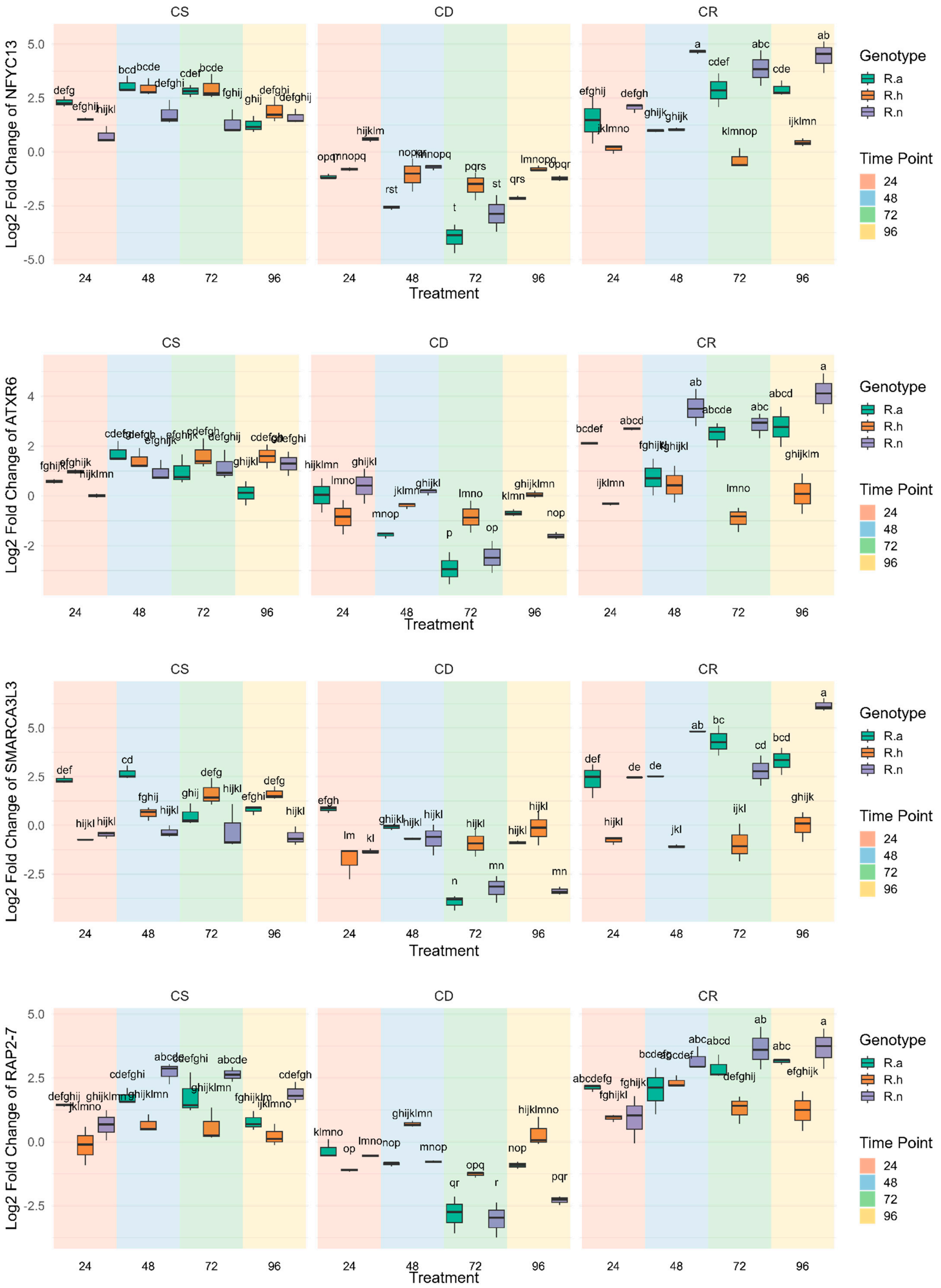
CS
CD
CR
Log2 Fold Change of NFYC13
Log2 Fold Change of ATXR6
Log2 Fold Change of SMARCA3L3
Log2 Fold Change of RAP2-7
Treatment
24
48
72
96
Genotype
R.a
R.h
R.n
Time Point
24
48
72
96

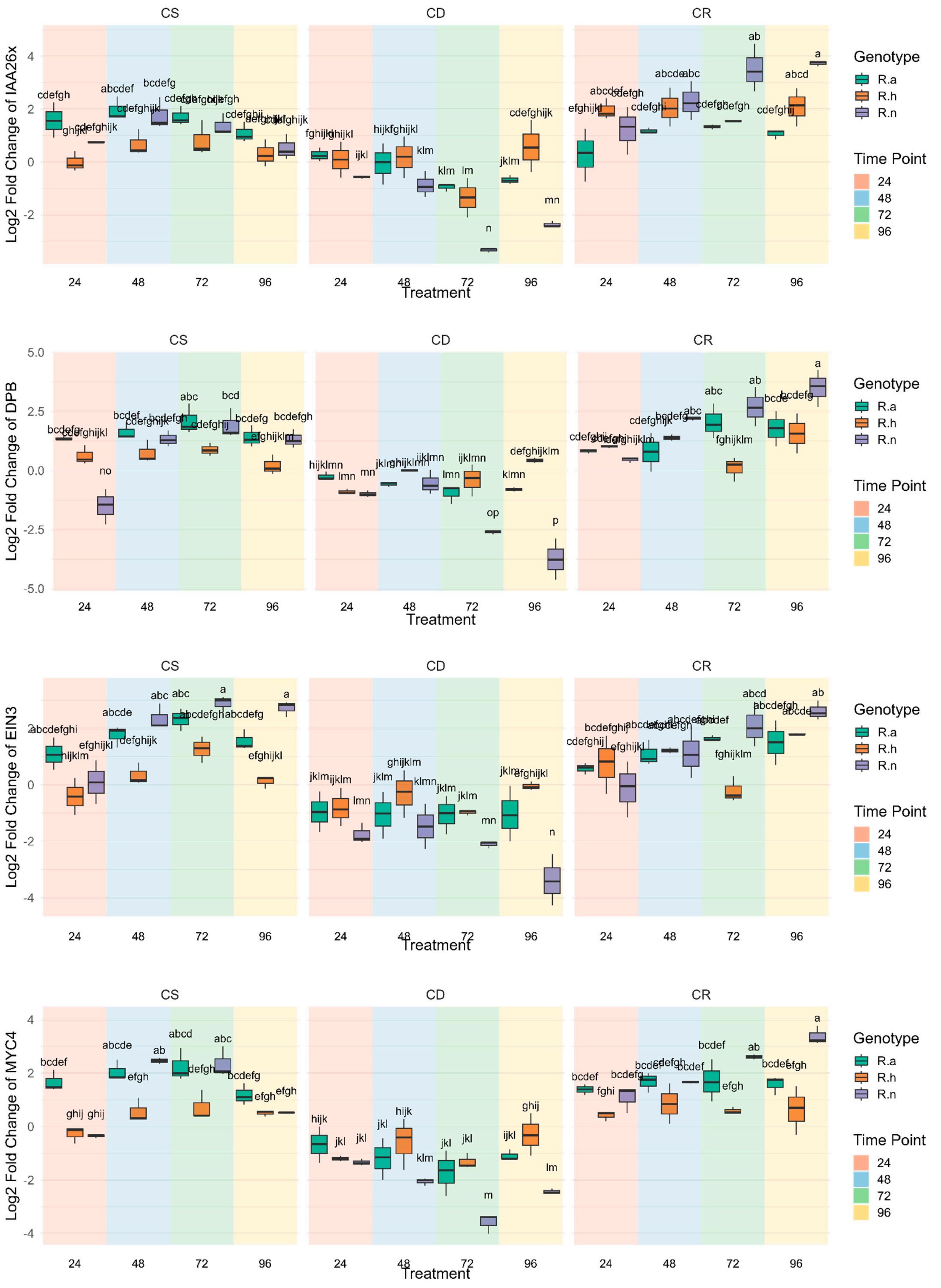
CS
CD
CR
Log2 Fold Change of IAA26x
Log2 Fold Change of DPB
Log2 Fold Change of EIN3
Log2 Fold Change of MYC4
Treatment
24
48
72
96
Genotype
R.a
R.h
R.n
Time Point
24
48
72
96

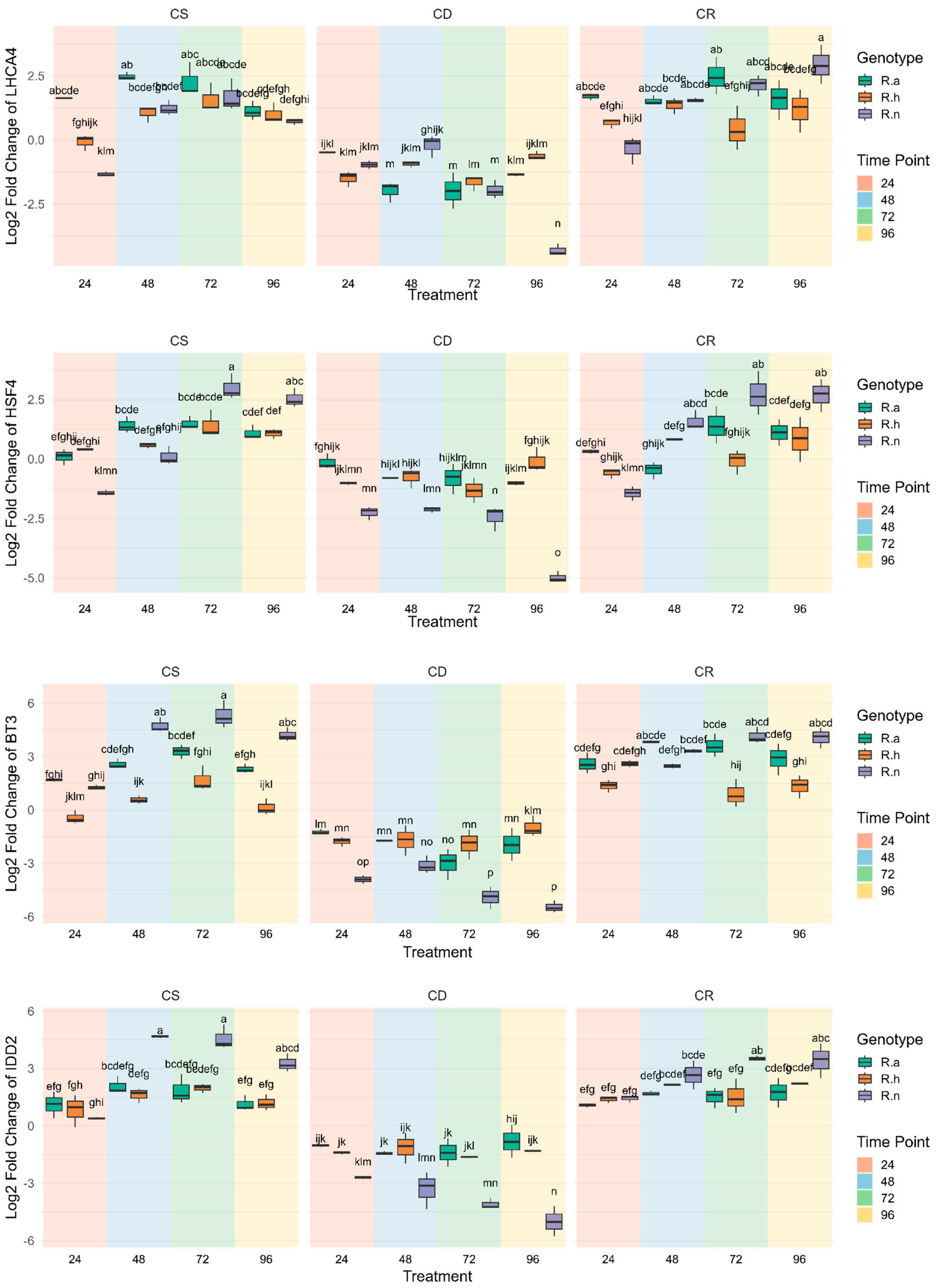
CS
CD
CR
Log2 Fold Change of LHCA4
Log2 Fold Change of HSF4
Log2 Fold Change of BT3
Log2 Fold Change of IDD2
24
48
72
96
Treatment
Genotype
R.a
R.h
R.n
Time Point
24
48
72
96

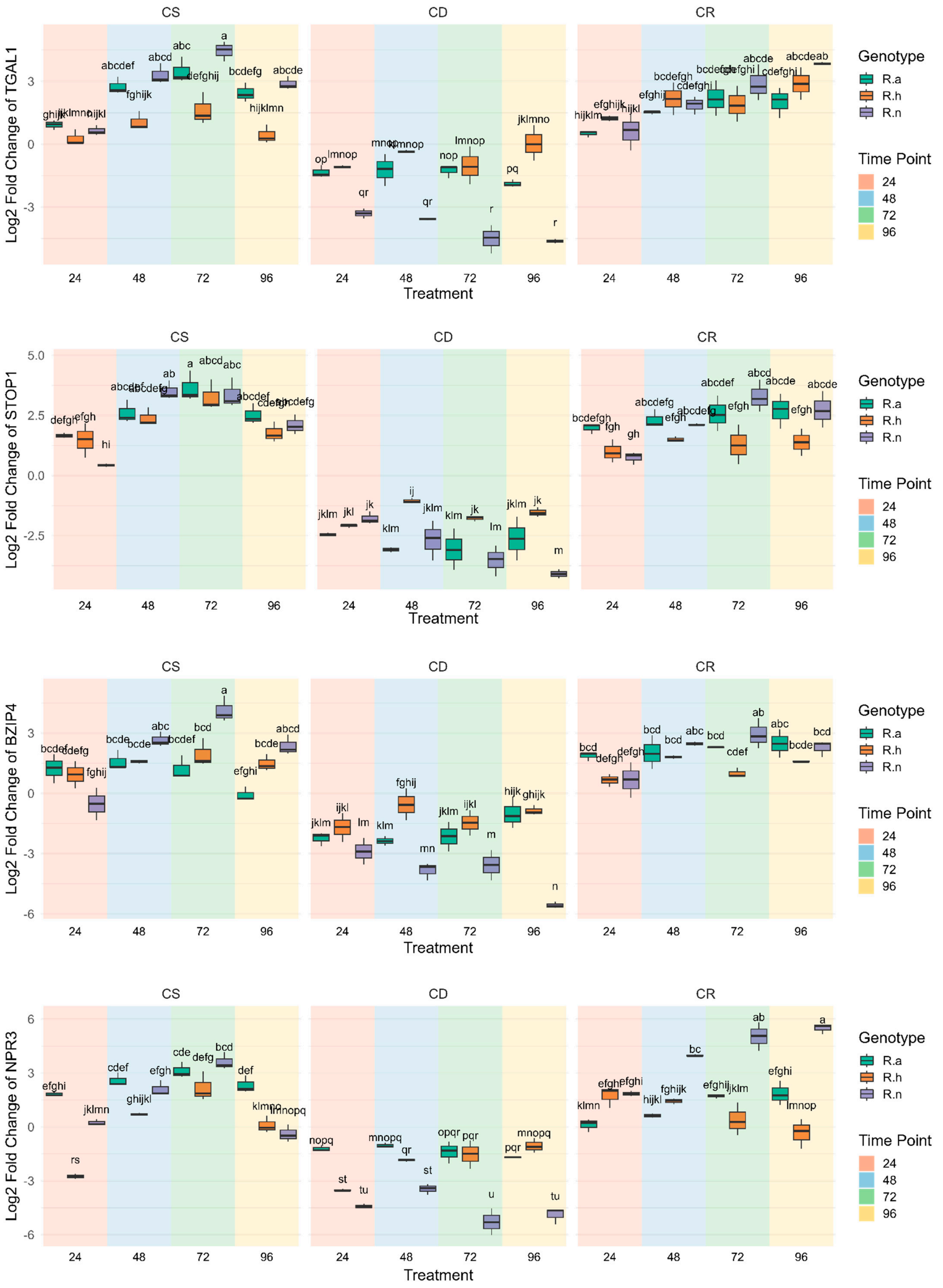
CS
CD
CR
Log2 Fold Change of TGAL1
Log2 Fold Change of STOP1
Log2 Fold Change of BZIP4
Log2 Fold Change of NPR3
Treatment
24
48
72
96
Genotype
R.a
R.h
R.n
Time Point
24
48
72
96

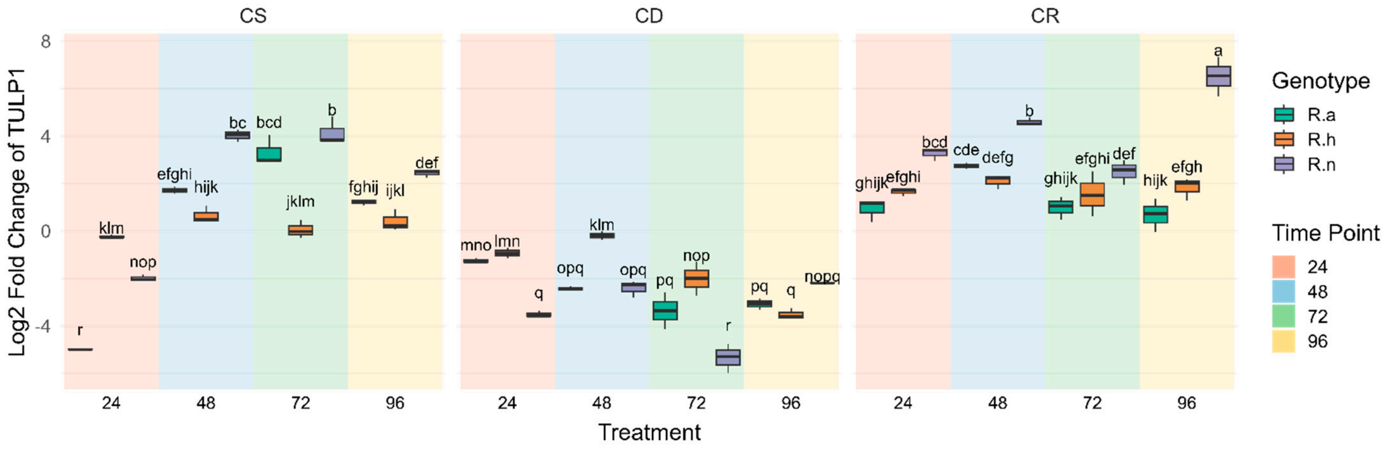

Figure S1. Boxplot of Log2 Fold Change of selected genes in *Ribes* species during cold stress, deacclimation, and reacclimation *in vitro* (n ≥ 3)**.** CS – cold stress, CD – cold deacclimation, CD – cold reacclimation; R.a – *R. aureum*, R.h – *R. hudsonianum*, R.n – *R. nigrum*; treatment period 24 – 96 h.
